# The Complete Genome Sequence of the Nicotine-Degrading Bacterium *Shinella* sp. HZN7

**DOI:** 10.3389/fmicb.2016.01348

**Published:** 2016-08-30

**Authors:** Jiguo Qiu, Youjian Yang, Junjie Zhang, Haixia Wang, Yun Ma, Jian He, Zhenmei Lu

**Affiliations:** ^1^Key Laboratory of Agricultural Environmental Microbiology, Ministry of Agriculture, College of Life Sciences, Nanjing Agricultural UniversityNanjing, China; ^2^Department of Microbiology, College of Life Sciences, Zhejiang UniversityHangzhou, China; ^3^Department of Environmental Science, College of Environment, Zhejiang University of TechnologyHangzhou, China

**Keywords:** *Shinella* sp. HZN7, PacBio, genome sequence, plasmids, nicotine, biodegradation, reference genome

## Background

Nicotine is a natural alkaloid that is very toxic to humans. To eliminate the harmful effects of nicotine in the environment, biological methods employing microbes to degrade nicotine are required (Brandsch, 2006; Liu et al., 2015). *Shinella* sp. HZN7 can degrade nicotine efficiently via the variant of a pyridine and pyrrolidine pathways (VPP; Ma et al., 2013; Qiu et al., 2014, 2015). The main intermediates in this pathway include 6-hydroxy-nicotine, 6-hydroxy-*N*-methylmyosmine, 6-hydroxypseudooxynicotine, 6-hydroxy-3-succinoyl-pyridine, and 2,5-dihydroxypyridine. This strain is the first nicotine-degrading bacterium to be isolated from the genus *Shinella*.

The genus *Shinella* was established in 2006 within the “*Rhizobiaceae* group” of the *Alphaproteobacteria* (An et al., 2006). Six species were assigned to this genus namely *S. daejeonensis, S. fusca, S. granuli, S. kummerowiae, S. yambaruensis, S. zoogloeoides*. However, most strains in this genus have not been identified at the species level. *Shinella* spp. have been isolated from various environmental samples, such as active sludge, zooplankton gut, soils, and water. They also exhibit a range of functional diversity, such as nitrogen fixation (Lin et al., 2008), assimilation of phosphite (Poehlein et al., 2016), and degradation of the toxic pollutants, 4-aminobenzenesulfonate (Biala et al., 2014), chlorothalonil (Liang et al., 2011), and pyridine (Bai et al., 2009). Until now, only three *Shinella* draft genomes have been deposited in GenBank (http://www.ncbi.nlm.nih.gov/genome/genomes/32494). To further understand the molecular mechanism of nicotine degradation and advance the potential biotechnological applications of *Shinella* strains, we present the first complete genome sequence of *Shinella* sp. HZN7 and its features.

## Materials and methods

### Bacterial strain and DNA purification

*Shinella* sp. HZN7 was isolated from the active sludge of a wastewater-treatment system of a pesticide manufacturer in Hangzhou City, China. This bacterium was cultured aerobically in LB medium at 30°C with 100 μg/mL ampicillin. Genomic DNA from *Shinella* sp. HZN7 was extracted and purified using a QIAamp DNA Mini Kit (Qiagen, Germany). The concentration of genomic Genomic DNA was measured using a Qubit 2.0 Fluorometer (Thermo Scientific, USA). Purity of DNAs samples (UV A_260_/A_280_) was assessed using a NanoDrop 2000 Spectrophotometer (Thermo Scientific, USA).

### Genome sequencing and assembly

The genome of strain HZN7 was sequenced using the PacBio RSII platform. A 20-kb DNA library was constructed according to the manufacturer's instructions and sequenced using single-molecule realtime (SMRT) sequencing technology with the P6 DNA polymerase and C4 chemistry. The sequences from two SMRT cells were assembled with SMRT Pipe version 2.1.1 using the hierarchical genome-assembly process (HGAP). The reads were *de novo* assembled and polished using the PacBio software HGAP3/Quiver (Chin et al., 2013).

### Genome annotation

The coding sequences (CDSs) were predicted using the Prokaryotic Genome Annotation Pipeline (PGAP) version 3.2 software on NCBI (https://www.ncbi.nlm.nih.gov/genome/annotation_prok/). The locus tag prefix was set as “*shn.*” Additional gene prediction and annotation was performed using the Rapid Annotation Subsystems Technology (RAST) server (Aziz et al., 2008). CRISPR finder (http://crispr.u-psud.fr/Server/) was used for identifying CRISPR/Cas systems (Grissa et al., 2007).

## Results

### Genome features

After quality control, ~4 Gb of data was obtained with a 550-fold average coverage. A total of 7,354,253 bp genome sequence was assembled. The features for the complete genome sequence of *Shinella* sp. HZN7 are summarized in Table 1. The complete genome is composed of one circular chromosome and 12 circular plasmids (designated as plasmid pShin-01 to pShin-12) with an average GC content of 64.8%. The whole genome contains 6954 genes, including 6694 coding sequences, 3 5S rRNAs, 3 16S rRNAs, 3 23S rRNAs, 51 tRNAs, 4 ncRNA, and 196 pseudo genes. Interestingly, 215 mobile genetic elements were predicted, including 96 transposases, 33 integrases, and 86 conjugative transfer proteins. Moreover, one CRISPR gene cluster was identified by the CRISPR finder tool. To the best of our knowledge, there are no bacterial strains containing up to 12 circular plasmids. Previous reports have shown that *Shinella zoogloeoides* strain BC026 contained 3 or more 200 kb megaplasmids (Bai et al., 2010) and *Shinella* sp. DD12 contained at least seven plasmids (Poehlein et al., 2016). These results indicate that the possession of multiple plasmids is a common feature in the genus *Shinella*.

**Table 1:** **Genomic features of ***Shinella*** sp. HZN7**.

**Features**	**Chromosome**	**pShin-01**	**pShin-02**	**pShin-03**	**pShin-04**	**pShin-05**	**pShin-06**
Genome size (bp)	4,678,597	620,539	445,803	409,126	222,555	155,026	147,896
G + C content (%)	65.2	66.2	65.5	65.3	65.1	58.5	62.1
Total genes	4480	571	389	386	200	144	149
Protein coding genes	4334	556	376	379	187	137	144
RNA genes	64	0	0	0	0	0	0
Mobile genetic elements	85	1	4	3	1	30	9
GenBank accession No.	CP015736	CP015737	CP015738	CP015739	CP015740	CP015741	CP015742
**Features**	**pShin-07**	**pShin-08**	**pShin-09**	**pShin-10**	**pShin-11**	**pShin-12**	**Total**
Genome size (bp)	132,822	128,968	127,666	112,708	100,630	71,917	7,354,253
G + C content (%)	60.6	66.0	61.7	61.3	60.6	59.9	64.8
Total genes	134	117	119	115	88	62	6954
Protein coding genes	125	110	105	106	82	53	6694
RNA genes	0	0	0	0	0	0	64
Mobile genetic elements	12	2	24	23	3	18	215
GenBank accession No.	CP015743	CP015744	CP015745	CP015746	CP015747	CP015748	

### Nicotine-degrading gene cluster

Our previous study showed that the novel 6-hydroxy-nicotine oxidase, NctB, was responsible for the degradation of 6-hydroxy-nicotine to 6-hydroxypseudooxynicotine (Qiu et al., 2014). The *nctB* gene (locus tag *shn_30305*) was found on the plasmid pShin-05. The *nctB* gene, as well as genes homologous to *vppA* (nicotine hydroxylase gene), *vppE* (2,5-dihydroxypyridine dioxygenase gene) from *Ochrobactrum* sp. strain SJY1 (Yu et al., 2015) and *pno* (6-hydroxypseudooxynicotine oxidase gene) from *Agrobacterium tumefaciens* S33 (Li et al., 2016), appeared in an 50 kb region of DNA with a GC content of 56.6%. The predicted genes (*shn_30145* to *shn_30370*) in this cluster and their characterized homolog are summarized in Table 2. This cluster was not found in three other *Shinella* draft genomes (http://www.ncbi.nlm.nih.gov/genome/genomes/32494). In addition, two transposase genes flanked this cluster of DNA, indicating that it may have been acquired by horizontal gene transfer.

**Table 2:** **Summary of gene cluster involved in nicotine degradation in ***Shinella*** sp. HZN7**.

**Gene locus tag**	**Size (amino acids)**	**Genes with predicted function**	**Function of most similar gene product(s)**	**Source accession no. and identity**
shn_30145	157	Transposase	–	–
shn_30195	391	Para-nitrophenol 4-monooxygenase	6-Hydroxy-3-succinoylpyridine 3-monooxygenase, VppD	AIH15770 (100%)
shn_30205	671	Hypothetical protein	6-Hydroxypseudooxynicotine oxidase, Pno	WP_024899819 (100%)
shn_30230	736	Chemotaxis protein	Methyl-accepting chemotaxis protein, MCP	Q00986 (47%)
shn_30235	344	Putrescine/spermidine ABC	–	–
		Transporter substrate-binding protein		
shn_30250	358	DDE endonuclease	–	–
shn_30255	226	Tetr family transcriptional regulator	–	–
shn_30260	344	Putrescine/spermidine ABC	–	–
		Transporter substrate-binding protein		
shn_30265	266	ABC transporter permease	–	–
shn_30270	301	ABC transporter permease	–	–
shn_30275	357	Spermidine/putrescine ABC	–	–
		Transporter ATP-binding protein		
shn_30280	210	Carbamoylsarcosine amidase	Maleamate amidase, VppG	AIH15798 (100%)
shn_30285	342	Leucyl aminopeptidase	2,5-DHP dioxygenase, VppE	AIH15799 (99%)
shn_30290	260	Alpha/beta hydrolase	*N*-formylmaleamic acid deformylase, VppF	AIH15800 (100%)
shn_30295	249	Asp/Glu racemase	Maleate isomerase, VppH	AIH15801 (100%)
shn_30300	465	Aldehyde dehydrogenase	4-Aminobutanal dehydrogenase	Q6D6Y7 (39%)
shn_30305	437	Hypothetical protein	6-Hydroxy-nicotine oxidase, NctB	AGS16700
shn_30310	551	Transposase	–	–
shn_30325	527	Hypothetical protein	Nicotine hydroxylase large subunit VppA_*L*_	AIH15806 (100%)
shn_30330	155	(2Fe-2S)-binding protein	Nicotine hydroxylase small subunit VppA_*S*_	AIH15807 (100%)
shn_30370	477	Transposase	–	–

–, experimental information is not available.

In conclusion, we present the first complete genome of *Shinella* sp. HZN7. We hope this will facilitate a deeper the understanding of the molecular mechanism of nicotine degradation via the VPP pathway, and provide a reference genome for genus *Shinella*.

## Ethics statement

This article does not contain any studies with human participants or animals performed by any of the authors.

## Data access

The complete genome sequence of *Shinella* sp. HZN7 has been deposited at the GenBank/EMBL/DDBJ under the accession numbers CP015736-CP015748. The strain is available from the China Center for Type Culture Collection under the accession no CCTCC M 2013060 or from Dr. JQ at Nanjing Agricultural University.

## Author contributions

JQ and ZL conceived and designed the research; YY, JZ, and HW performed experiments and analyzed data; JQ, YM, JH, and ZL analyzed data; JQ and ZL wrote the manuscript; all authors commented on the manuscript and approved the contents.

### Conflict of interest statement

The authors declare that the research was conducted in the absence of any commercial or financial relationships that could be construed as a potential conflict of interest.
